# Using the Internet to Teach Health Informatics: A Case Study

**DOI:** 10.2196/jmir.3.3.e26

**Published:** 2001-09-29

**Authors:** David Parry, Alec Holt, John Gillies

**Affiliations:** ^1^Business FacultyAuckland University of TechnologyAucklandNew Zealand; ^2^Department of Information ScienceUniversity of OtagoDunedinNew Zealand; ^3^Wellington School of MedicineWellingtonNew Zealand

**Keywords:** Medical Informatics, Education, Distance, Internet, Universities, New Zealand

## Abstract

**Background:**

It is becoming increasingly important for health professionals to have an understanding of health informatics. Education in this area must support not only undergraduate students but also the many workers who graduated before informatics education was available in the undergraduate program. To be successful, such a program must allow currently-employed students with significant work and family commitments to enroll.

**Objectives:**

The aim was to successfully create and teach a distance program in health informatics for the New Zealand environment.

**Methods:**

Our students are primarily health professionals in full time employment. About 50% are doctors, about 25% nurses, and the rest include dentists, physiotherapists, and medical managers. Course material was delivered via the World Wide Web and CD-ROM. Communication between students and faculty, both synchronous and asynchronous, was carried out via the Internet.

**Results:**

We have designed and taught a postgraduate Diploma of Health Informatics program using the Internet as a major communication medium. The course has been running since July 1998 and the first 10 students graduated in July 2000. About 45 students are currently enrolled in the course; we have had a dropout rate of 15% and a failure rate of 5%. Comparable dropout figures are hard to obtain, but a recent review has suggested that failure-to-complete rates of 30% to 33% may be expected.

**Conclusions:**

Internet technology has provided an exciting educational challenge and opportunity. Providing a web-based health informatics course has not been without its frustrations and problems, including software compatibility issues, bandwidth limitations, and the rapid change in software and hardware. Despite these challenges, the use of Internet technology has been interesting for both staff and students, and a worthwhile alternative for delivering educational material and advice to students working from their own homes.

## Introduction

Health informatics is not just information science for health professionals. One definition of health or medical informatics is that it: "Comprises the theoretical and practical aspects of information processing and communication, based on knowledge and experience derived from processes in medicine and health care." [1] It has been said that: "Computers are to health informatics, what stethoscopes are to cardiology." [2] However, computers remain an essential part of any teaching about health informatics. As Edward Shortliffe has said: "Schools need to look beyond computer literacy concerns to develop formal informatics curricula that meet the needs of future practitioners who will function as users and creators of data and information." [3] Internet technology is starting to be used in the medical-education process in Australia and New Zealand and, increasingly, information-technology skills are needed for good medical practice. This paper addresses issues relating to the use of the Internet in teaching health informatics.

The Internet was chosen as a major medium for delivery of the course because New Zealand has a long and narrow shape and a sparse population. Although distances between centers are not great, travel in New Zealand is inconvenient, especially for the people who have been identified as likely to participate in the Diploma Course. A distance method of teaching is needed to allow more potential students to take part. As Hersh [4] states, potential students for courses like this are likely to be working, have limited time, and be unwilling to move to complete a traditional course. In particular, the authors and other members of the development team believe that the use of Internet communication would significantly increase the quality of the educational experience of the students, allowing better communication between participants and better support for students.

## Methods

The Course [5] is aimed at increasing the number of people skilled in health informatics in New Zealand. Although there is significant interest in the topic, health workers in New Zealand have uneven skill levels and often work outside their own personal skill level in isolated environments. The primary goals of the course are:

- Raising health professionals' understanding of health informatics and computer technology, including: the effective use of common software, communication tools, and some of the concepts underlying the use of computers in health care.
- Providing academic recognition of informatics skills, by awarding a diploma.
- Developing a network of expertise, comprising alumni and the current students and staff, to promote informatics in the New Zealand health system.
- Reaching people working in relatively-remote environments.

The developers of the course chose to deliver the course by electronic methods, especially because these methods are flexible and the students need to receive and work on the course material at times that suit their lifestyle.

The diploma is one of a number offered by the University of Otago, the second-largest university in New Zealand. Entry is open to graduates with at least a 3-year bachelors degree and to people with equivalent education (such as nurses trained before the bachelor-of-nursing degree was introduced) who are working in the field of healthcare or have a strong interest in it. Fees vary but are about US $4000 for the whole diploma for New Zealand residents. Until now, advertising for this course has been confined to New Zealand.

Students have participated from all of the provinces of New Zealand and from overseas (Australia, Brunei, Papua New Guinea, and the United Kingdom) but all face-to-face meetings have occurred in New Zealand. Our students are primarily health professionals in full-time employment. About 50% are doctors and about 25% are nurses; the rest include dentists, physiotherapists, and medical managers. The students study outside of regular working hours. Because the need for significant travel would preclude students' participation in a traditional face-to-face course, the authors and the development team hoped the use of Internet technologies for communication would increase the participation and interest of the students in the course, as well as giving the students an insight into the use of these communication technologies, which are rapidly becoming vital tools of health professionals.

The authors and the development team believe that this form of education is likely to be widely used in the future and that the benefits of electronic teaching include:

- Demonstration of the advantages of instruction using electronic media such as: hyperlinks, multimedia, and all the tools that computer-based courses can provide.
- Potential increase in the students' confidence in their ability to learn new software packages, based on their use of the course material, which is a software package, albeit one that is designed to be easy to use.
- Advice on the most suitable software applications from a teaching staff experienced in this form of course delivery.
- A flexible working pattern for the students, with support for different learning styles [4].

The authors met weekly via Internet technologies while developing the educational material - and continue to meet weekly using these technologies. In addition, the authors have met face-to-face. The authors use Internet technologies for direct supervision of the students.

A number of communication software packages have been tested and evaluated. We expect to continue to try out new technologies as they appear, and will use newer ones when they offer superior reliability, flexibility, or both.

**Overview of the Diploma of Health Informatics Course**

The New Zealand Education University system includes a period of study called a "paper." Points for a paper are based on the hours of study per 15-week semester. Each point is roughly equivalent to 2 hours of study per week. For this diploma, the student must obtain 40 points to graduate. To earn 40 points, a student must complete and pass 4 papers, since each paper with a passing (above 50%) grade is worth 10 points and no points are awarded for a failed paper.

The course includes 2 compulsory introductory papers, each lasting 15 weeks. Students are sent a questionnaire before the course to assess their level of computing expertise. They are then classified into 1 of 3 groups based on level of expertise (Table 1).

Students have rarely had any programming experience and have rarely previously completed any university-level courses in computing. Even students scoring at quite high levels of expertise are interested and stimulated by the introductory paper, especially by learning the techniques required for group interaction.

**Table 1:** Expertise levels of students, based on skills

**Expertise level**	**Skills**
Advanced	programs from the Internet or CD-ROM
Medium	Office Professional software
Beginner	Has experience with word processing. Can use e-mail

The initial paper (701) is an introduction to the computer and to standard office-suite software. Microsoft Office Professional is used as the teaching tool, along with graphics packages such as Paint Shop Pro and HTML-development tools such as Composer or FrontPage. Although the students may have used these packages before, we expect the students to be able to gain a deeper understanding of the use of these tools and ultimately to develop simple programs using Visual Basic for Applications. The second paper (702) provides an overview of the discipline of health informatics.

There are 5 other papers, from which the student selects 2 to complete the requirements for the diploma. These papers examine in more detail specific aspects of health informatics. The full list is:

- 701 Essential information-management skills
- 702 Principles of health informatics
- 703 Project development and software design
- 704 Evidence-based practice
- 705 Computer-aided learning
- 706 Research methods and statistics in the health sector
- 707 Personal project in the area of health informatics

For each paper, each student is given: a CD-ROM with the main course material, a list of recommended textbooks, and a reading list with between 20 and 30 journal articles or book chapters.

**Figure 1:**
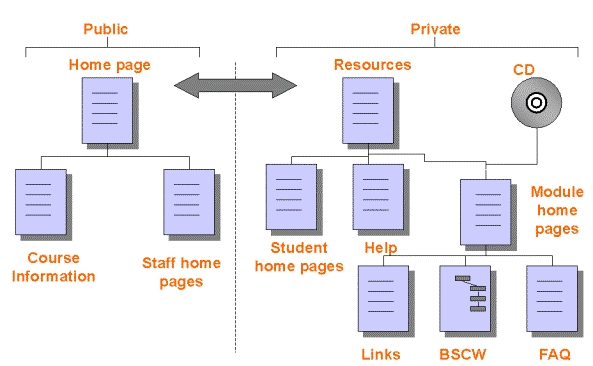
Communication links and electronic resources for the diploma of health informatics

Other relevant material, including hyperlinks to more resources, is posted on a Web-based file-sharing system, Basic System for Cooperative Work (BSCW) [6],. BSCW provides areas for asynchronous discussion, file storage, and hyperlinks, with an extensive and powerful security model that allows rights to be assigned to individual users and groups. Asynchronous discussion is similar to discussion by e-mail except that all related postings are grouped together in "threads," as is the case in Usenet newsgroups. For example, a student may post a question, and both staff and students may reply to it. Because communication is asynchronous, these replies may be spread over a number of days. Interested people can view the progress of the discussion and contribute to it at times of their choosing. In contrast, synchronous discussion is similar to a discussion by telephone, requiring that all parties are present at the same time. Some areas of BSCW are set up to allow posting of files and hyperlinks by staff, some by students, and some by both. Details of the electronic resources, including BSCW, are shown in Figure 1. BSCW is used primarily for material and files that are updated during the semester; at the end of the semester, this material is archived and, if appropriate, included on the Web-site or CD-ROM the next time the paper is offered. The World-Wide-Web-based materials in the private area are password protected. Errata and additional materials to support the CD-ROM are also published in the private area.

### Details of Teaching Techniques

#### Workshop

The authors consider a face-to-face workshop that is held at the beginning of all papers essential to the effective functioning of the participants after they have returned to their own working environment. When participants interact from their remote sites, each participant requires a mental picture of other participants. Initially, attendance at these workshops was not compulsory, but non-attendees found difficulty in participating fully in the group discussions, so this attendance was made compulsory. For these workshops, subsidized travel and accommodation is provided for students. Until now, all workshops have been held in New Zealand, but if overseas participation increases, workshops may be held overseas in, for example, the United Kingdom.

The goals of the workshops are for the students to:

- Meet and interact with fellow participants and the teaching staff.
- Start work on their group projects (described below, in Coursework and Communication), with face-to-face meetings.
- Learn about the communications software (described below, in Coursework and Communication).
- Learn about aspects of the course and meet experts in the field.
- Have fun.

In the first workshop, the aim is to provide the student with a thorough background in use of the conferencing software and the BSCW server. Most students arrive at the workshop feeling a little unsure about their capabilities; it is vital that the students gain confidence while still at the workshop, as this significantly reduces technical problems once they have returned home. In addition to practical demonstrations and exercises on their own, we provide a printed handbook for each participant.

#### Course Materials

**Figure 2:**
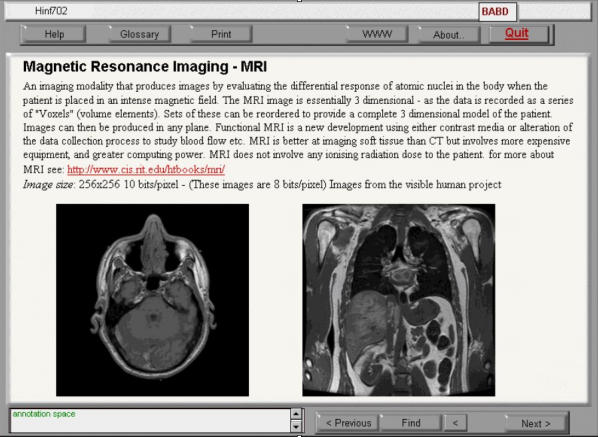
Screen shot from a course CD-ROM

At the workshop, students are given a CD-ROM containing the course materials, along with a paper booklet of photocopied readings. The CD-ROM was constructed using Macromedia Authorware 5.1 and includes graphics, text, and videos (Figure 2). Students can save annotations to the CD-ROM on their own machines' hard drive; and a log is kept on their machine of the "pages" they have visited. The CD-ROM includes software needed for the course (eg, for zipping/unzipping [compressing/uncompressing files] and for browsing), a printable PDF (Portable Document Format) image of the course material, and extra example-application files. Each CD-ROM has about 10 modules, with a competency test for each module (see below, in Coursework and Communication). A textbook such as van Bemmel's book [1] is supplied for each paper.

#### Coursework and Communication

Of the total marks assigned in each paper, 60% are given for individual tests and 40% are given for a group project.

The group project is an interesting and exciting challenge for all participants, including the staff members. In each paper, there is a group project that aims to reinforce and expand the topics discussed in that paper. Group projects have resulted in reports subsequently published in this journal [7]. In addition to the group projects students submit competency tests, for example databases or Web-pages they have developed, at set times during the semester. The group meetings (described in more detail later in this section) are also used as tutorials and discussions about the current competency test. Although this imposed timetable reduces flexibility, it allows students with problems to be identified early in the semester, before they are too far behind.

After the initial work in the face-to-face meetings at the workshop, the group meets using Internet-conferencing software. The authors and development team have tried synchronous packages and asynchronous packages, and each group decides upon the package, or combination of packages most suitable for their needs. Most choose NetMeeting and e-mail as their primary communication methods and BSCW as their location for posting documents. Where there is difficulty with the Internet-based audio-conferencing such as NetMeeting some groups have elected to use one of a variety of text-chat software options (eg, ICQ).

Internet-based audio-conferencing is presently difficult to manage, and the technology is often unreliable. We have established a set of guidelines for running Internet-based audio-conferences, to maximize the benefits of time spent on-line. Students become frustrated when they lose contact with the meeting or when they cannot hear; at present, these are recurring issues with Internet-based audio-conferences. Computer hardware is important to reliability, since some sound cards are not fully compatible with the software. The card must have full-duplex sound capability (the ability to support sound transmission and reception at the same time), to allow efficient communication. A 56K or faster modem allows much higher quality of communication.

The guidelines for running Internet-based meetings are:

- Participants must have met in person.
- Agenda is prepared and circulated in advance.
- Chairperson controls meeting and writes minutes.
- Method of voting is agreed on - eg, it is agreed that silence equals consent.
- Chairperson summarizes at the end of each agenda topic.
- Minutes are circulated the same day as the meeting.

### Asynchronous communication tools.

**Figure 3:**
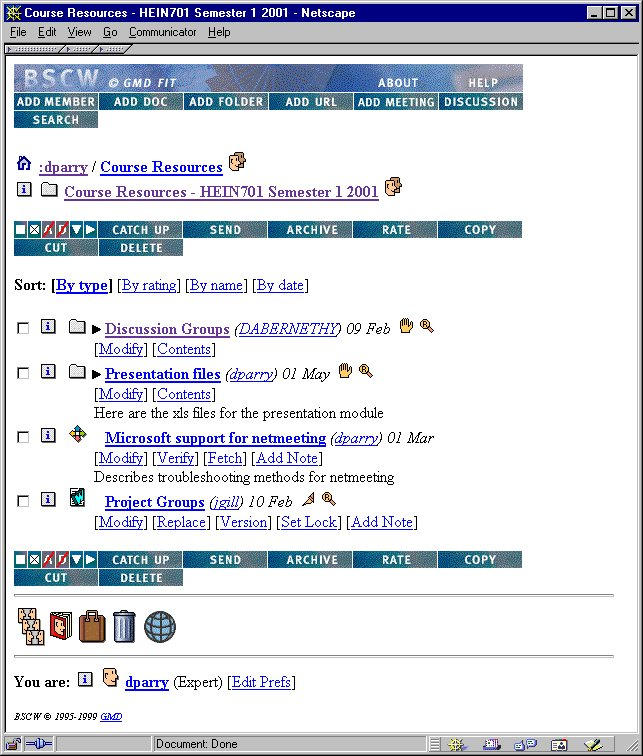
BSCW screen shot

The majority of the asynchronous work was done using e-mail which, for the University of Otago system, is limited to messages less than or equal to 1 Megabyte (MB). Although we set up some newsgroups, the majority of document sharing and threaded discussions took place via BSCW, BSCW was developed by the German Institute for Information Technology [8] and is free for use by educational institutions. BSCW is a collection of scripts written in the Python programming language that reside on a World Wide Web (WWW) server and allow secure file storage that is accessed via Web pages. One of the most useful features of BSCW is the ability to "version" a document. This allows changes to be made, while still allowing access to the original document and intermediate versions. Access control allows the staff to limit what each user of BSCW can see - for example, discussion groups, a software archive, and the user's project area -with appropriate rights of access (for example, read, write, or delete). The teaching staff has access to all areas including the definitive course-production documents, student results, and administrative areas. A typical BSCW screen shot is shown in Figure 3.

We are running the Windows NT version of BSCW 3.2, using Microsoft's Internet Information Server 4.

### Synchronous communication tools.

**Table 2:** Assessment of synchronous communication tools

**Tool Name (Tool Company)**	**Tool Type**	**Number of Participants**	**Cost**	**Degree of Use**	**Comments**
VoxChat (Voxware)	Audio chat and text chat; half-duplex*	Up to 5	Free	Used by all staff and students when the course was launched	longer supported
Internet Conference Professional (VocalTec)	Audio chat and text chat, whiteboard†	No limit imposed by software, but needs 4 KB/second bandwidth per audio connection to server (eg, for 10 users the server's connection to the Internet must be at least 40 KB/second of bandwidth)	About US $40 per seat‡ plus an unknown cost for server	Widely used, when first purchased; subsequent use forestalled by licensing problems	Easy-to-use and reliable product let down by a complicated mode for connecting to a meeting; no longer supported
ConferenceRoom (WebMaster)	Text chat	Up to 200	US $100	Used as a backup system if other systems not available	A simple-to-use, Web-based text chat system, with few features
NetMeeting (Microsoft) and OnLive server (White Pine)	Text chat and audio chat, whiteboard†, application sharing	5 free; up to 25 with licensed server	OnLive (the server): US $2,500 for 25 seats‡ for educational use;NetMeeting (the client): free	Used by all groups since 1999	OnLive extends NetMeeting by allowing audio chat between more than 2 participants
ICQ (Miribalis)	Text chat	No limit	Free	Used by 1 group	Easy-to-use and well-designed

^*^ Half-duplex: user has to wait until sound reception stops before user can transmit sound.

^†^ Whiteboard: a common graphical area that can be seen and edited by all participants.

^‡^ Each computer requires a seat

We assessed some synchronous communication tools (Table 2), and have a policy of exploring new technologies as they become available. We appraised each system based primarily on cost, number of participants, and reliability. Currently we primarily use the NetMeeting/OnLive combination.

## Results

We have designed and taught a Postgraduate Diploma of Health Informatics program using the Internet as a major communication medium [5]. The first 10 students graduated from the course in July 2000. About 45 students are currently (July 2001) enrolled in the course because we have had a dropout rate of 15%, and a failure rate of 5%. Comparable dropout figures are hard to obtain, but a recent review has suggested that failure-to-complete rates of 30-33% may be expected [9]. Dropouts have tended to occur either just after the workshop, when fees can be refunded, or due to outside events, such as change in employment or family circumstances. Gender distributions and percentages of students qualified as a doctor are shown in Table 3. (Date-of-birth data is not presented because it is not recorded in our system.)

Student evaluation of the course, using the standard University of Otago evaluation system (a 5-point scale; 1 is worst, 5 is best) has averaged above 4 for satisfaction; the University-wide average is about 3.5. Unfortunately, this evaluation system does not allow comparison with other papers offered by the University.

We hope to make the papers available to a wider audience, both as a diploma and as a series of short courses. Initially this is being pursued in collaboration with the University of Tasmania and the University of Auckland. In addition, the 701 and 702 papers form the basis for some undergraduate teaching at the Wellington School of Medicine. Four of the diploma graduates are currently studying towards Masters degrees in health informatics and 1 is continuing on to a PhD. Some publications, for example [7], have been produced by students and staff as part of the course.

**Table 3:** Student Gender and Professional Background, up to June 2001

**Paper**	**Cumulative Number of Students**	**Female%**	**Male%**	**Medical Degree%**
701 (Introduction; compulsory)	65	43.1	56.9	43.1
702 (Overview; compulsory)	54	40.7	59.3	44.4
703 (Database Systems)	14	28.6	71.4	64.3
704 (Evidence Based Practice)	12	25.0	75.0	66.7
705 (Computer Aided Learning)	10	70.0	30.0	30.0
706 (Research Methods)	14	64.3	35.7	21.4
707 (Research Project)	6	50.0	50.0	66.7
				
**All papers**	**175**	**43.4**	**56.6**	**45.1**

Technical difficulties were encountered in 3 main areas:

1. **Software compatibility.** Although the course was based on the Windows platform, many of the synchronous communication tools had problems with different hardware and driver configurations. This was a particular problem with sound cards. Because the students used home PCs there was little control of the hardware setup or of other software that could cause potential problems. Even use of a standard office suite caused problems with different installations, especially when the software was pre-installed.
2. **Bandwidth limitations.** At the beginning of the course, a 33K modem was the standard for home use. Although 56K modems are now commonplace, many areas of New Zealand have difficulty obtaining connections at data transfer rates above 4 KB/second, because of telephone-line quality. In addition, the university network is chronically congested despite regular upgrades. This has often limited use of the audio tools for discussion. Files larger than 100 KB are routinely zipped to reduce bandwidth overheads.
3. **Software and Hardware Obsolescence.** This is a major issue due to the current lifetime of software. Since the course has started there have been 2 generations of Windows operating systems and 3 generations of Microsoft Office suites. Although changes from generation to generation are often cosmetic, changes in, for example, screen appearance, menu location, or menu labels impact strongly on the usefulness of videos or labeled diagrams that demonstrate particular actions needed to perform a task. File formats have also changed; this presents particular problems for Microsoft Access, as the earlier versions cannot read later files. Generally, older versions of software are not available after new versions are released, so the approach of sticking with an old version for a long time is not feasible.

These technical difficulties cause greater problems when students first begin work for the diploma than later on. As the students become more confident and skilled, they can overcome these difficulties, and indeed see this as one of their greatest achievements.

## Discussion

Teaching an electronic course requires a shift in attitude from teaching in a face-to-face environment. The majority of the interaction with the students is done via text-based methods, so the communication style is different from that of a classroom situation. Care has to be taken to avoid confusion, and feedback from the students has to be encouraged to confirm understanding. The delay in feedback with asynchronous communication can cause frustration and in some cases irritation. The deadlines for competency tests are on a Wednesday, so that students with difficulties working over the weekend can contact staff and receive a reply before the assignment is due.

We have used a combination of different packages to teach our course. It seems that at present this approach is superior in cost and flexibility to an approach based on a single solution [10]. Other courses in health and medical informatics (for example, those from Monash University in Melbourne, Australia [11] and Oregon Health Sciences University, USA [12]) have used techniques similar to ours although as graduate certificates they require less study than a diploma. The recent announcement that MIT is to make large amounts of material freely available on the WWW [13] suggests to us that course materials are not the only part of a course that has value to students. The announcement implies confidence on MIT's behalf that students will continue to enroll in the courses offered, and pay the substantial fees, for the numerous benefits of participation in a course as compared with solo study.

The overwhelming impression we have gained from our students is the enthusiasm they have for this subject and the method of teaching. Despite many technical difficulties, this teaching approach seems to succeed in inspiring the participating health professionals. As Internet methods become more reliable and potential students more accustomed to using them, these techniques will be available for wider continuing professional education and even for undergraduate training. With the increase in biomedical research and information, the demand for "just-in-time" learning is increasing, and Internet-based learning is one of the few practical methods of supplying this to professionals that do not have easy access to face-to-face instruction.

In the future, we hope to:

- Shift more course material from the CD-ROM to the Internet, possibly by using distributed databases to store the material, both on our server and on the student's hard drive.
- Apply these techniques to other subjects.
- Expand the course, through alliances to other countries.
- Use intelligent information processing to support students better - especially in terms of setting up the communication tools; for example, by automatically calculating the student's connection speed when they use the synchronous tools and by creating a database of frequently asked questions that can be searched using natural language queries.

For those wishing to set up a course on similar lines there is a wide range of sources of information; an excellent book is "Learning Networks: A Field Guide to Teaching and Learning Online" [14]. The Association for the Advancement of Computing in Education (AACE) [15] publishes a number of useful journals. The journal Academic Medicine [16] produces a yearly list of advanced projects using new technologies.

## Abbreviations

AACE: Association for the Advancement of Computing in Education
BSCW: Basic System for Cooperative Work
ICQ: I Seek You
KB: Kilobyte
MB: Megabyte
PDF: Portable Document Format
WWW: World Wide Web

## Multimedia Appendix

Downloadable slides

## Multimedia Appendix

Video (large file!): Use of BSCW
