# Supplementary material for: Using the Internet to Teach Health Informatics: A Case Study
Source: J Med Internet Res. 2001 Sep 29;3(3):e26. doi: 10.2196/jmir.3.3.e26 (PMC1761904; doi:10.2196/jmir.3.3.e26)
Supplement: Supplementary file 1 [file jmir_v3i3e26_app1.ppt]

## Slide 1
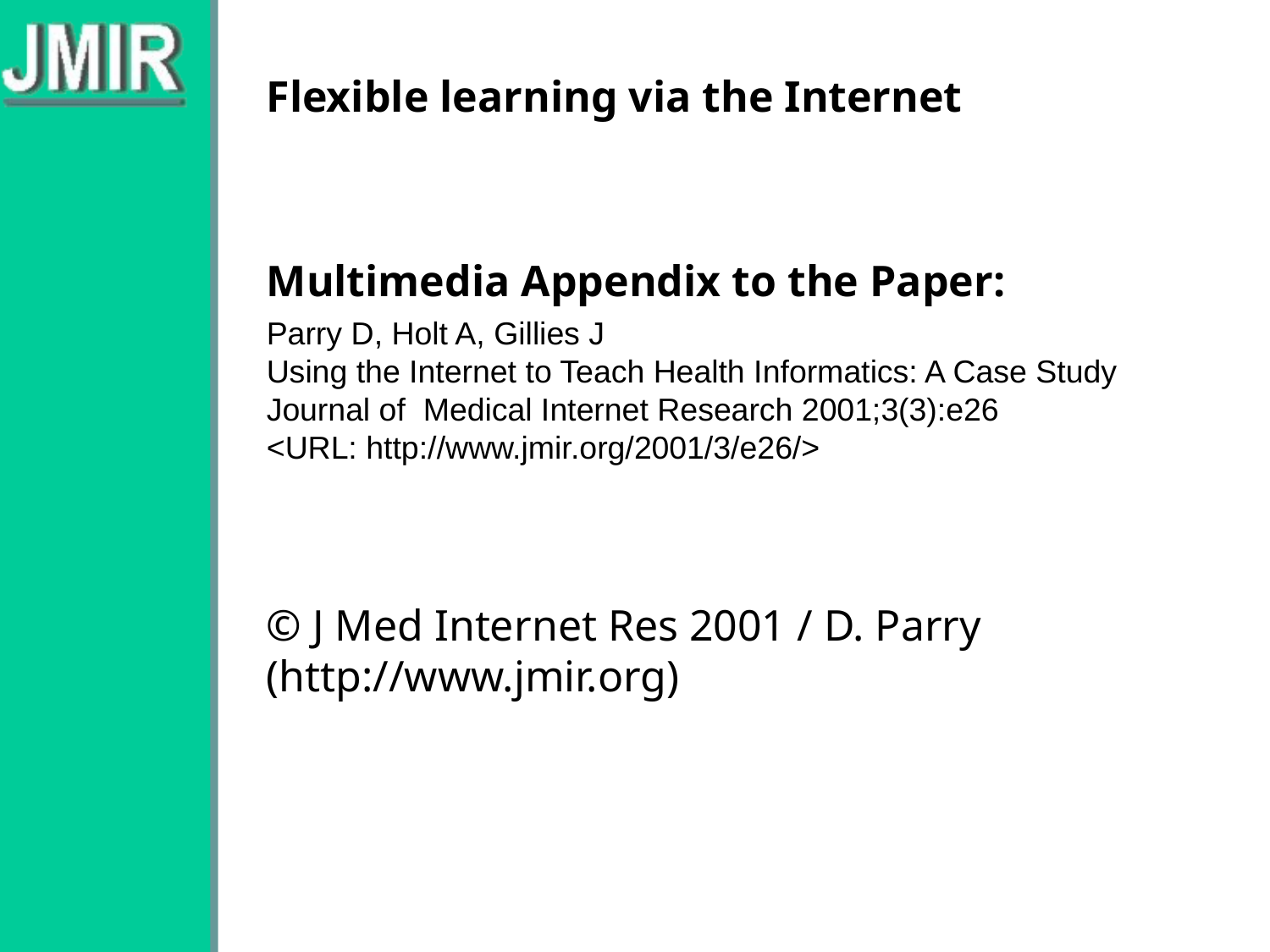

# Flexible learning via the Internet
Multimedia Appendix to the Paper:
Parry D, Holt A, Gillies J
Using the Internet to Teach Health Informatics: A Case Study
Journal of  Medical Internet Research 2001;3(3):e26
<URL: http://www.jmir.org/2001/3/e26/>
© J Med Internet Res 2001 / D. Parry (http://www.jmir.org)

## Slide 2
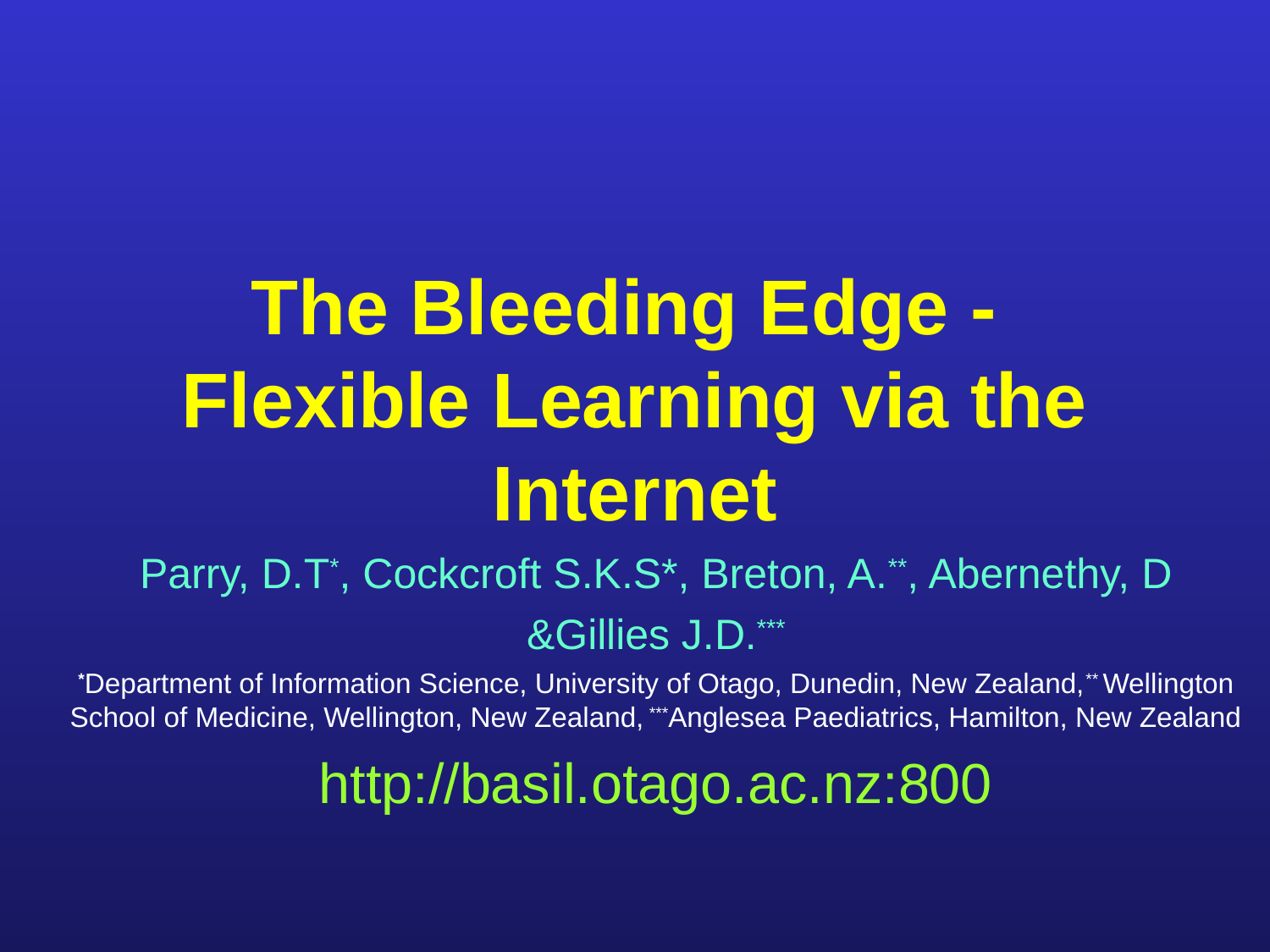

# The Bleeding Edge - Flexible Learning via the Internet
Parry, D.T*, Cockcroft S.K.S*, Breton, A.**, Abernethy, D
&Gillies J.D.***
*Department of Information Science, University of Otago, Dunedin, New Zealand,** Wellington School of Medicine, Wellington, New Zealand, ***Anglesea Paediatrics, Hamilton, New Zealand
http://basil.otago.ac.nz:800

## Slide 3
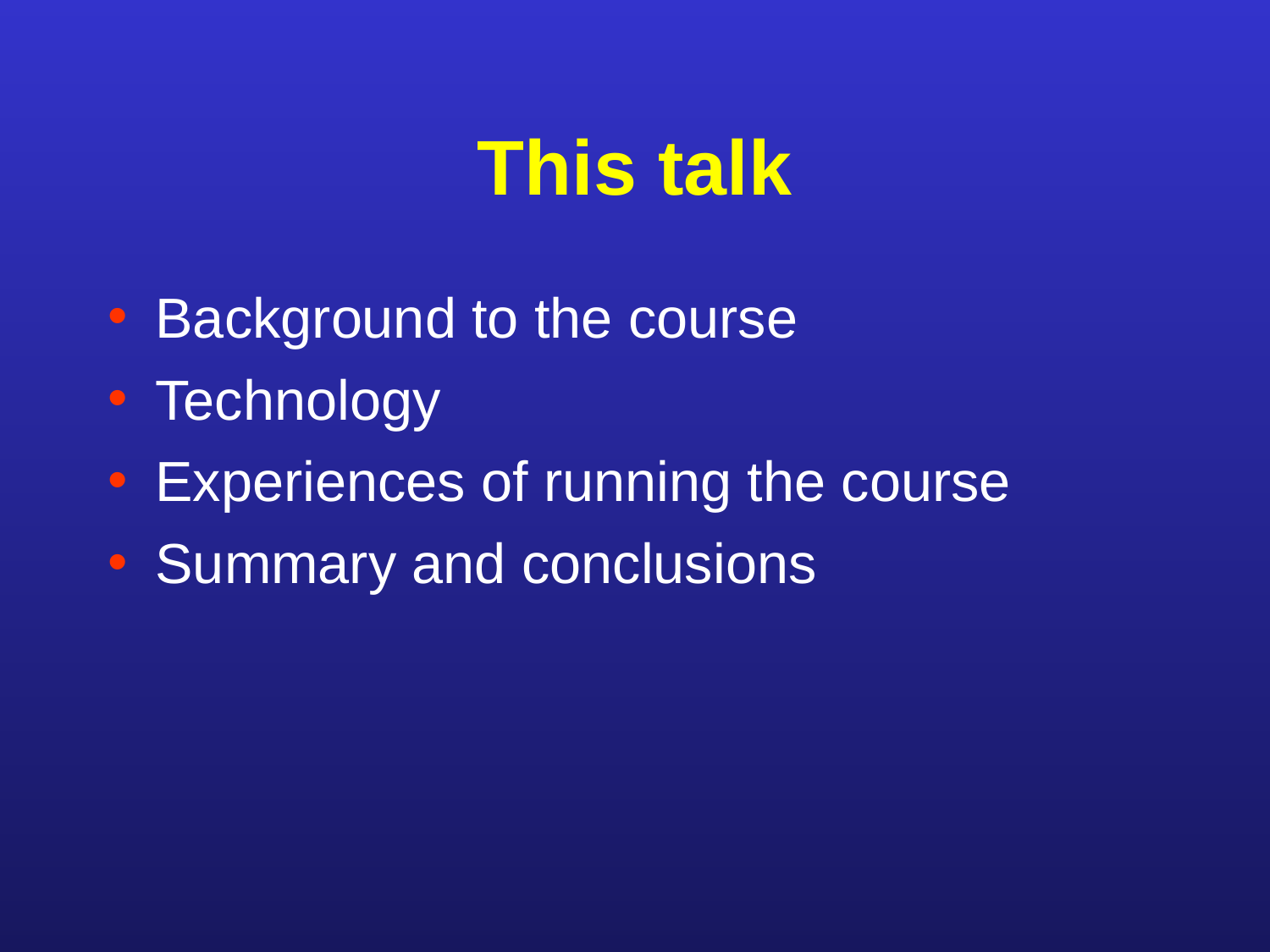

# This talk
Background to the course
Technology
Experiences of running the course
Summary and conclusions

## Slide 4
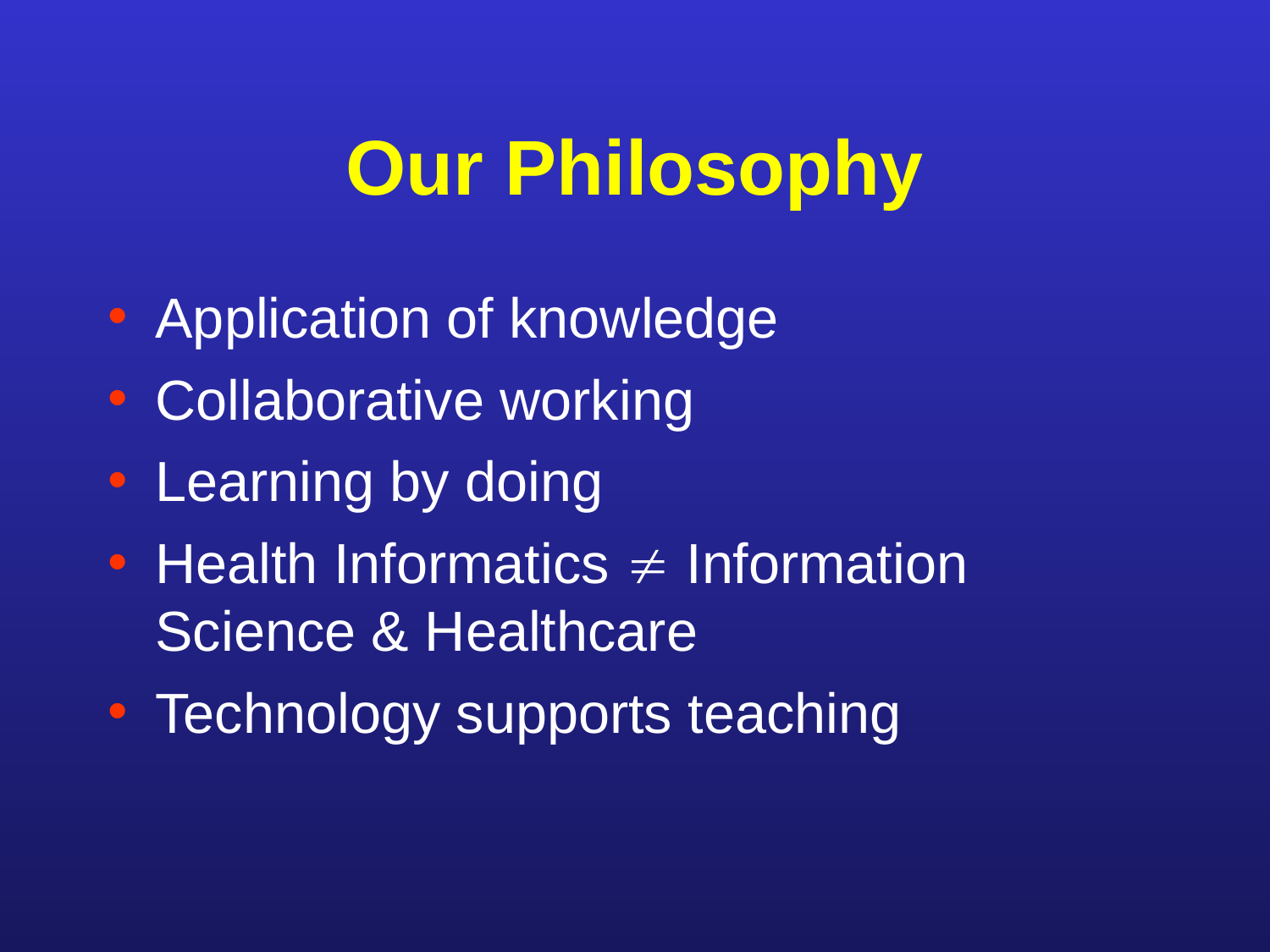

# Our Philosophy
Application of knowledge
Collaborative working
Learning by doing
Health Informatics  Information Science & Healthcare
Technology supports teaching

## Slide 5
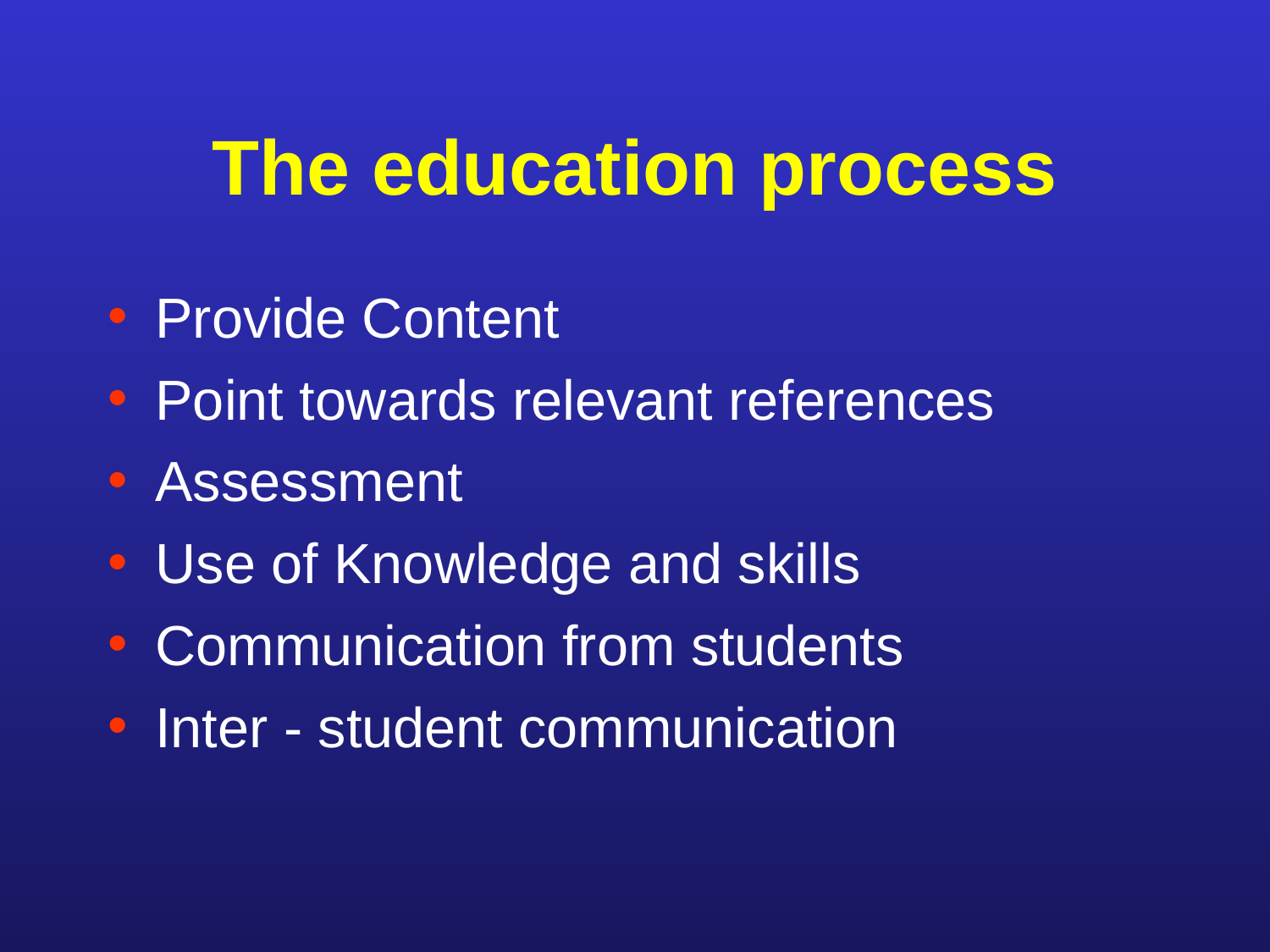

# The education process
Provide Content
Point towards relevant references
Assessment
Use of Knowledge and skills
Communication from students
Inter - student communication

## Slide 6
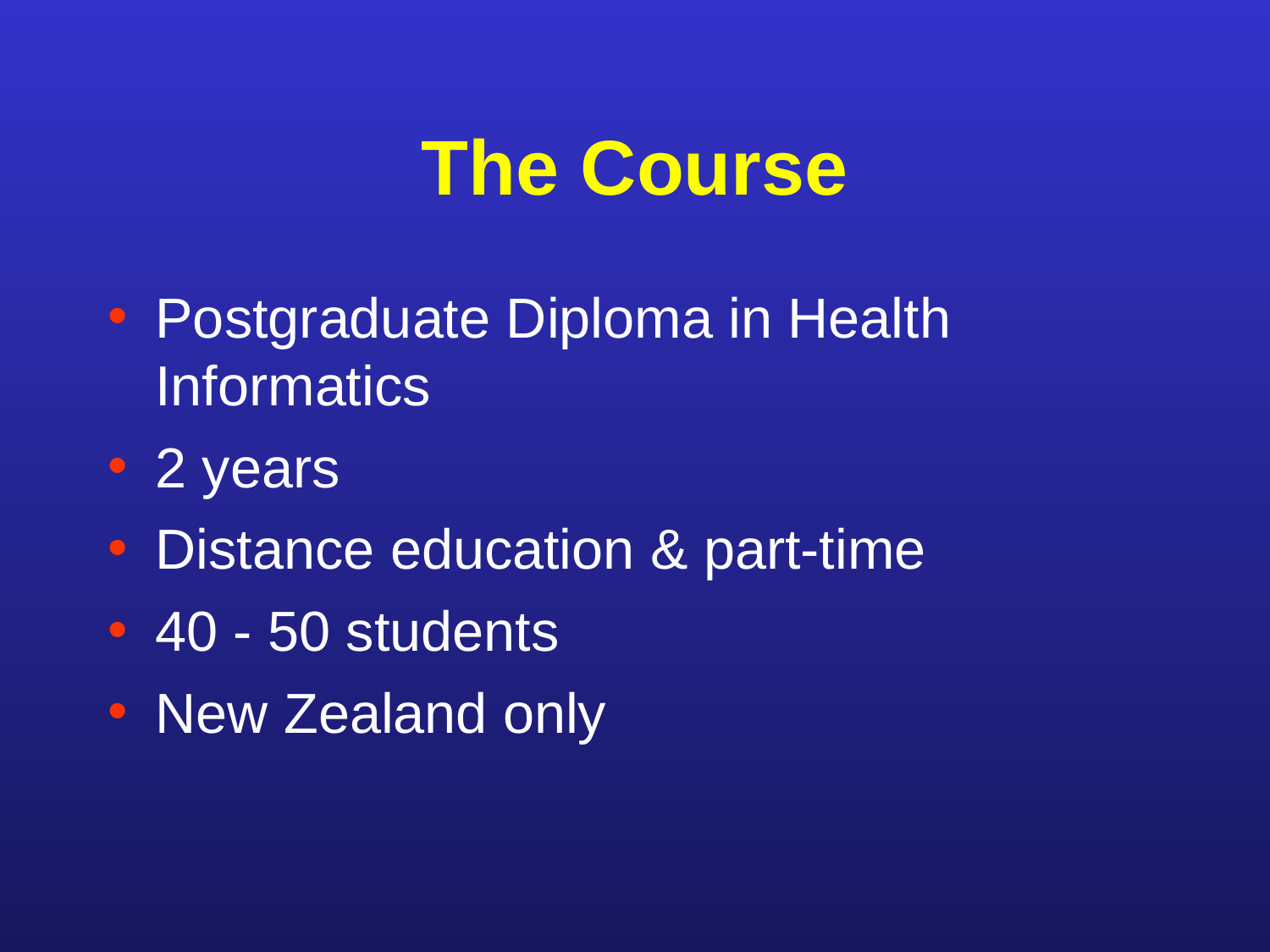

# The Course
Postgraduate Diploma in Health Informatics
2 years
Distance education & part-time
40 - 50 students
New Zealand only

## Slide 7
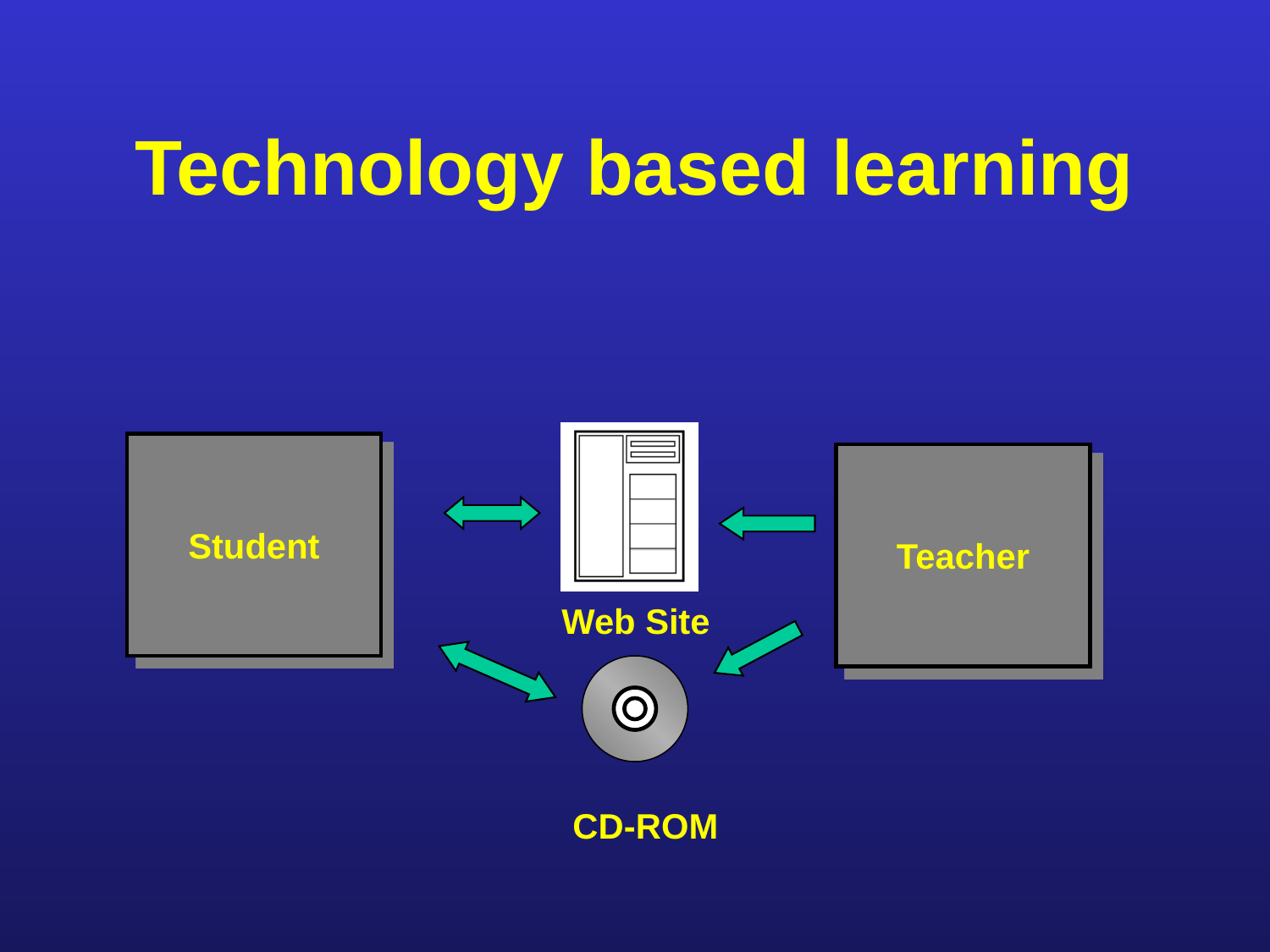

# Technology based learning
Student
Teacher
Web Site
CD-ROM

## Slide 8
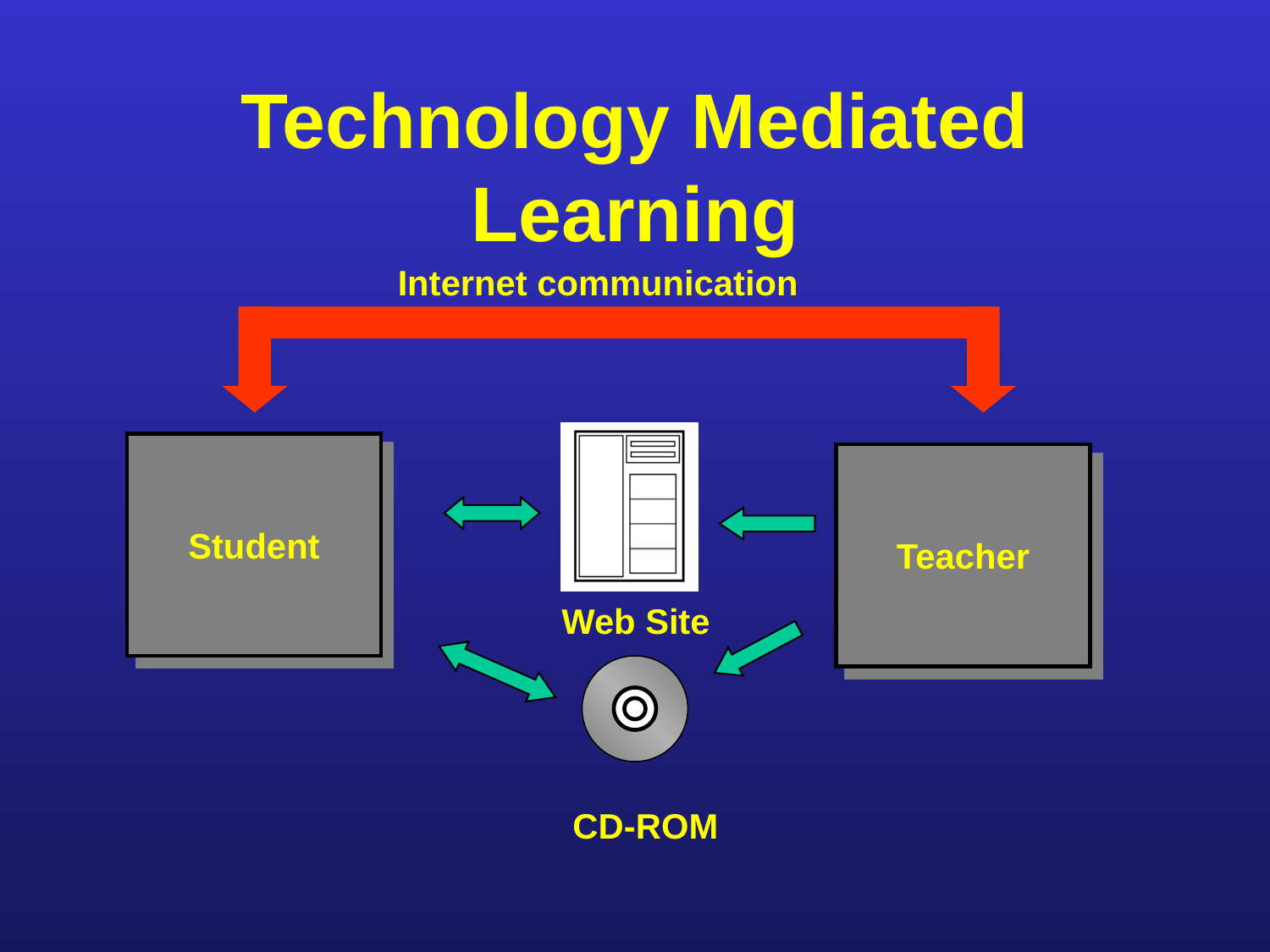

# Technology Mediated Learning
Internet communication
Student
Teacher
Web Site
CD-ROM

## Slide 9
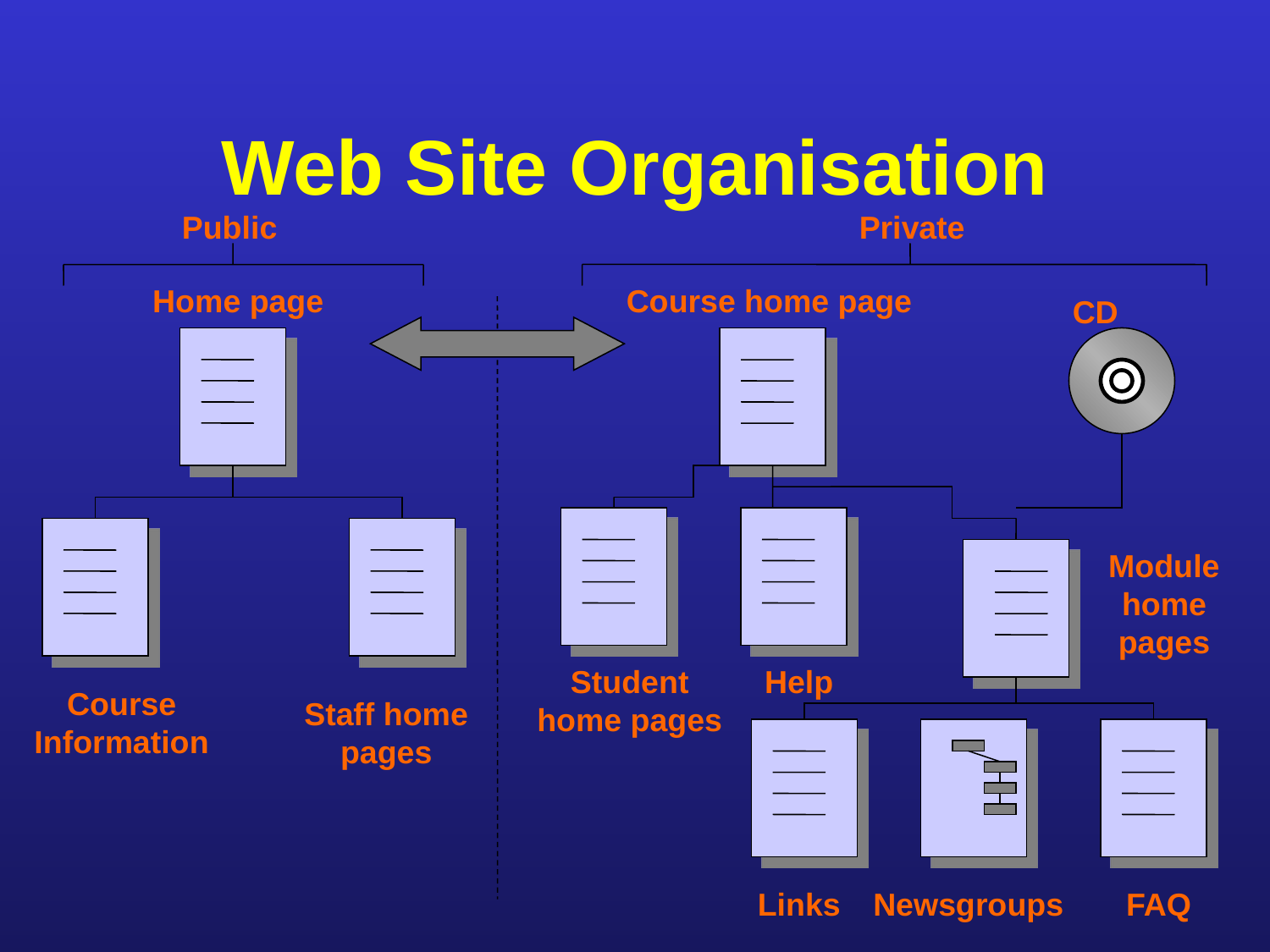

# Web Site Organisation
Public
Private
Home page
Course home page
CD
Module home pages
Student home pages
Help
Course Information
Staff home pages
Links
Newsgroups
FAQ

## Slide 10
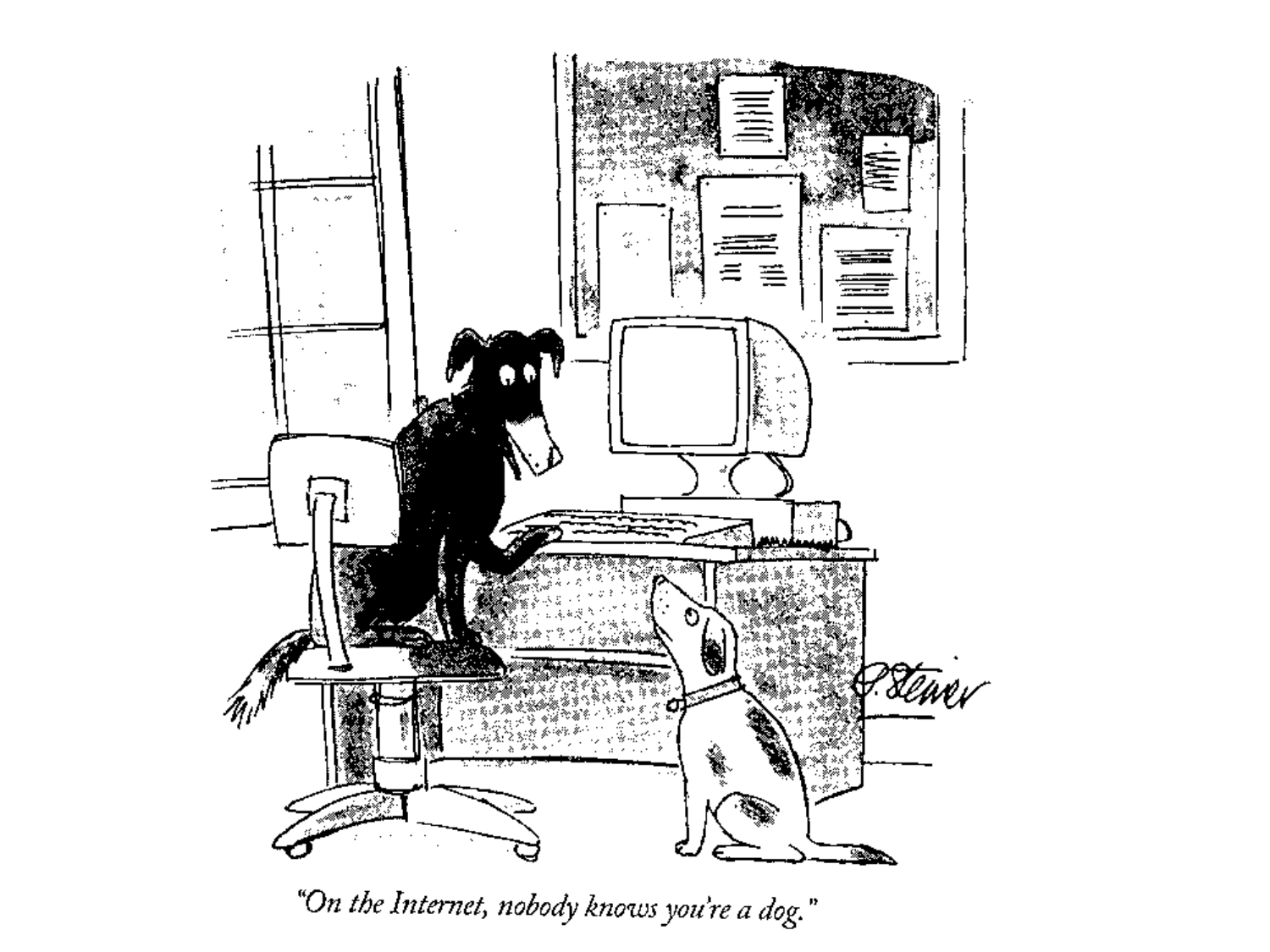

#

## Slide 11
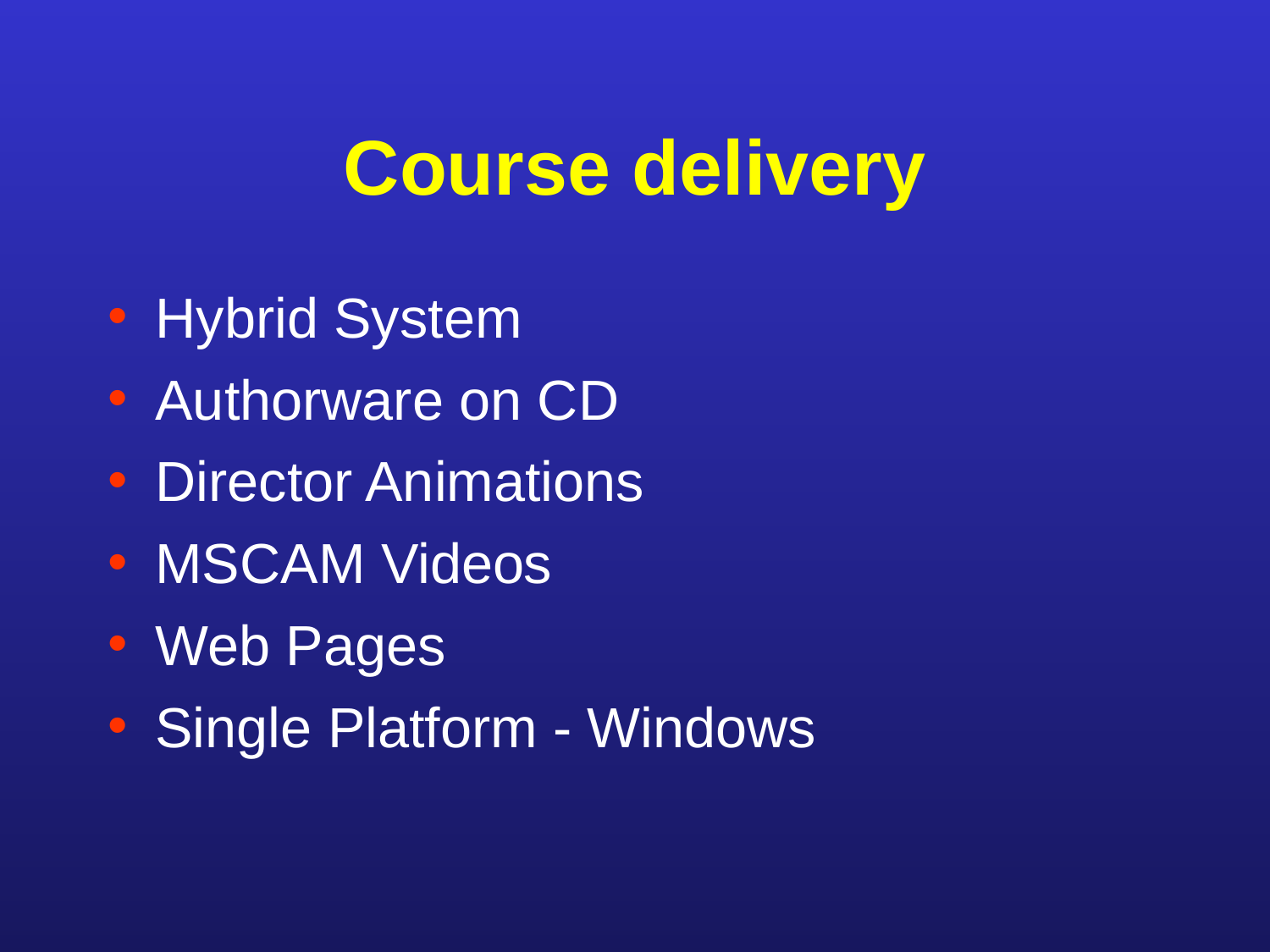

# Course delivery
Hybrid System
Authorware on CD
Director Animations
MSCAM Videos
Web Pages
Single Platform - Windows

## Slide 12
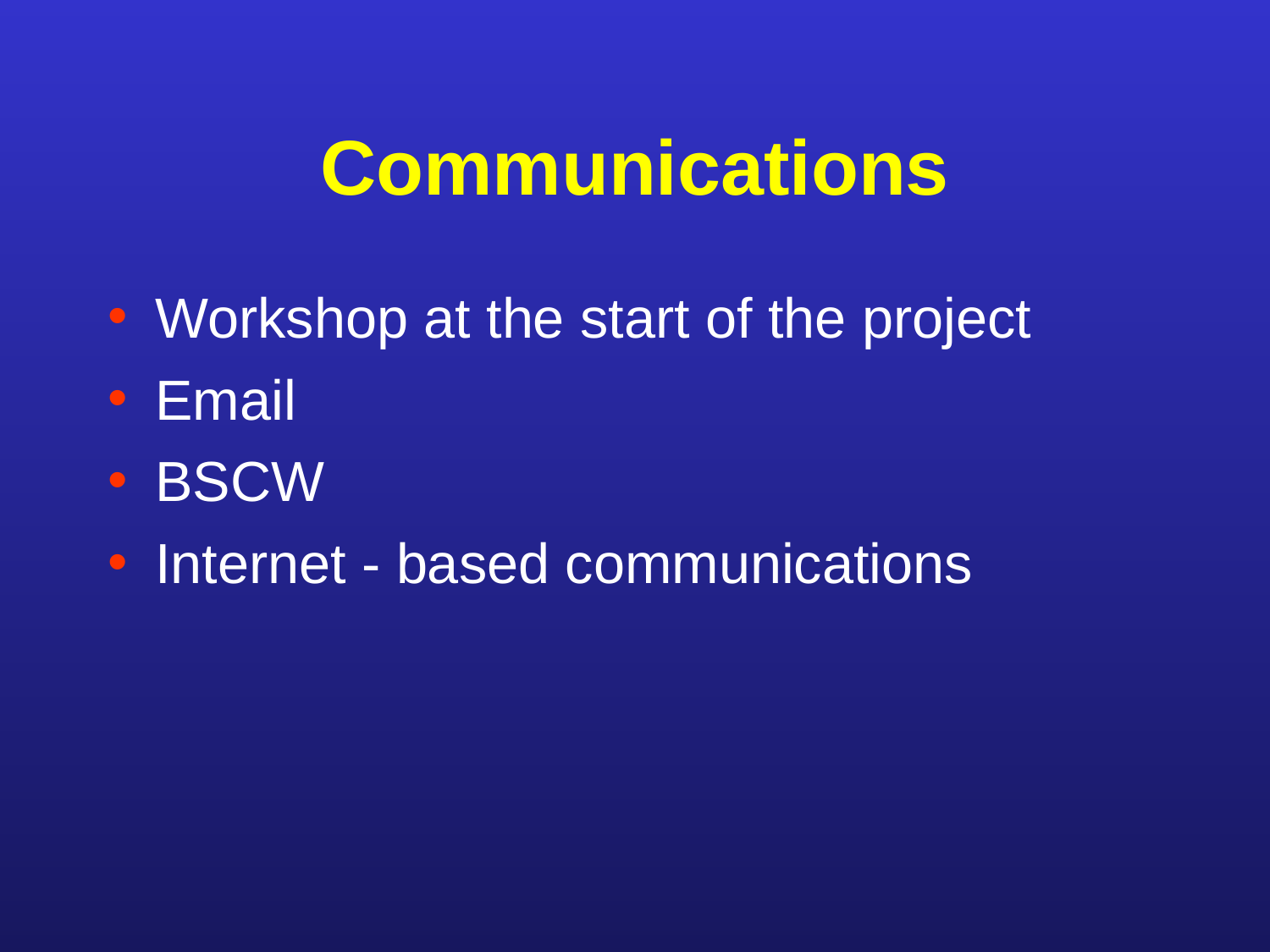

# Communications
Workshop at the start of the project
Email
BSCW
Internet - based communications

## Slide 13
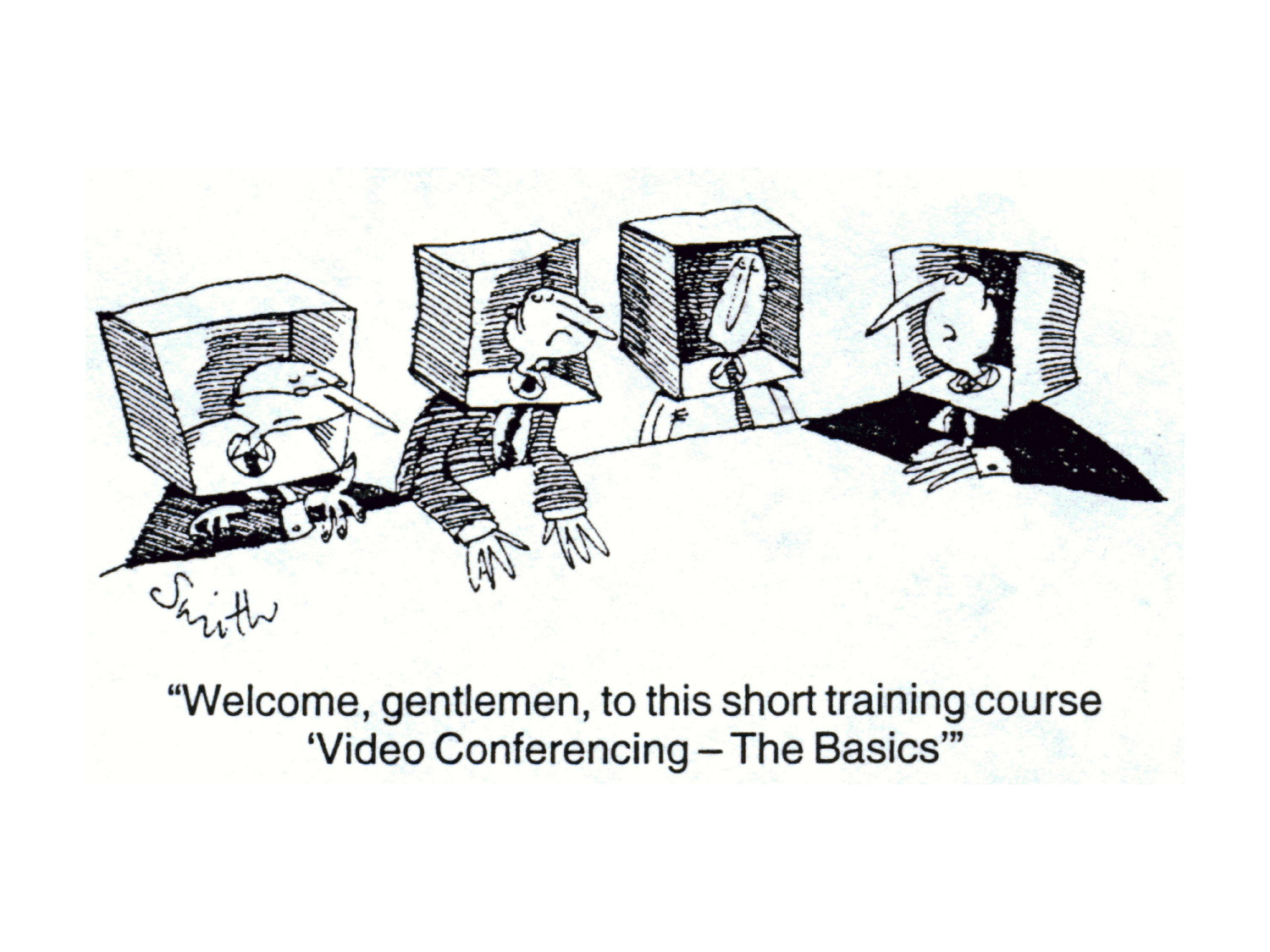

## Slide 14
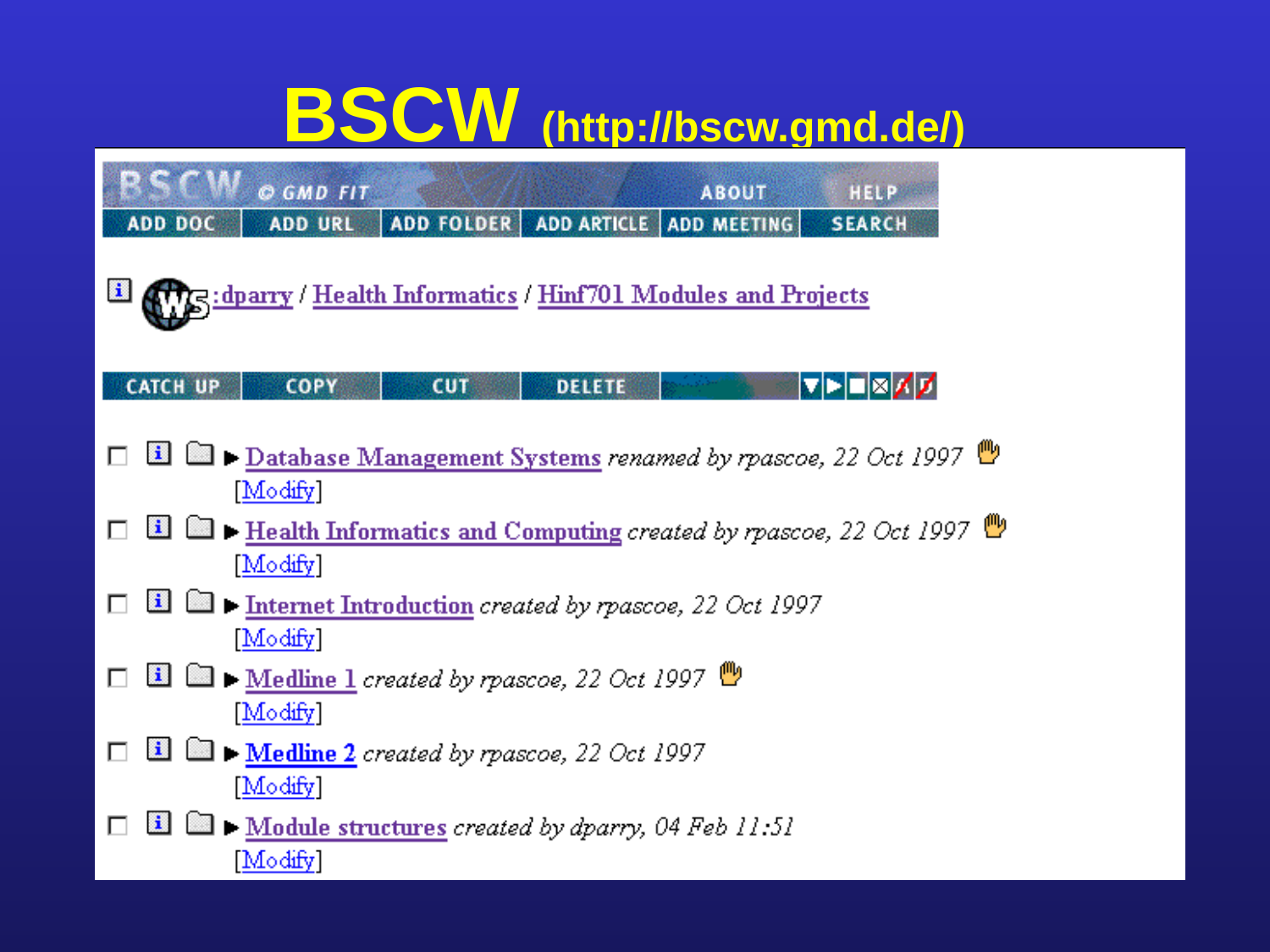

# BSCW (http://bscw.gmd.de/)

## Slide 15
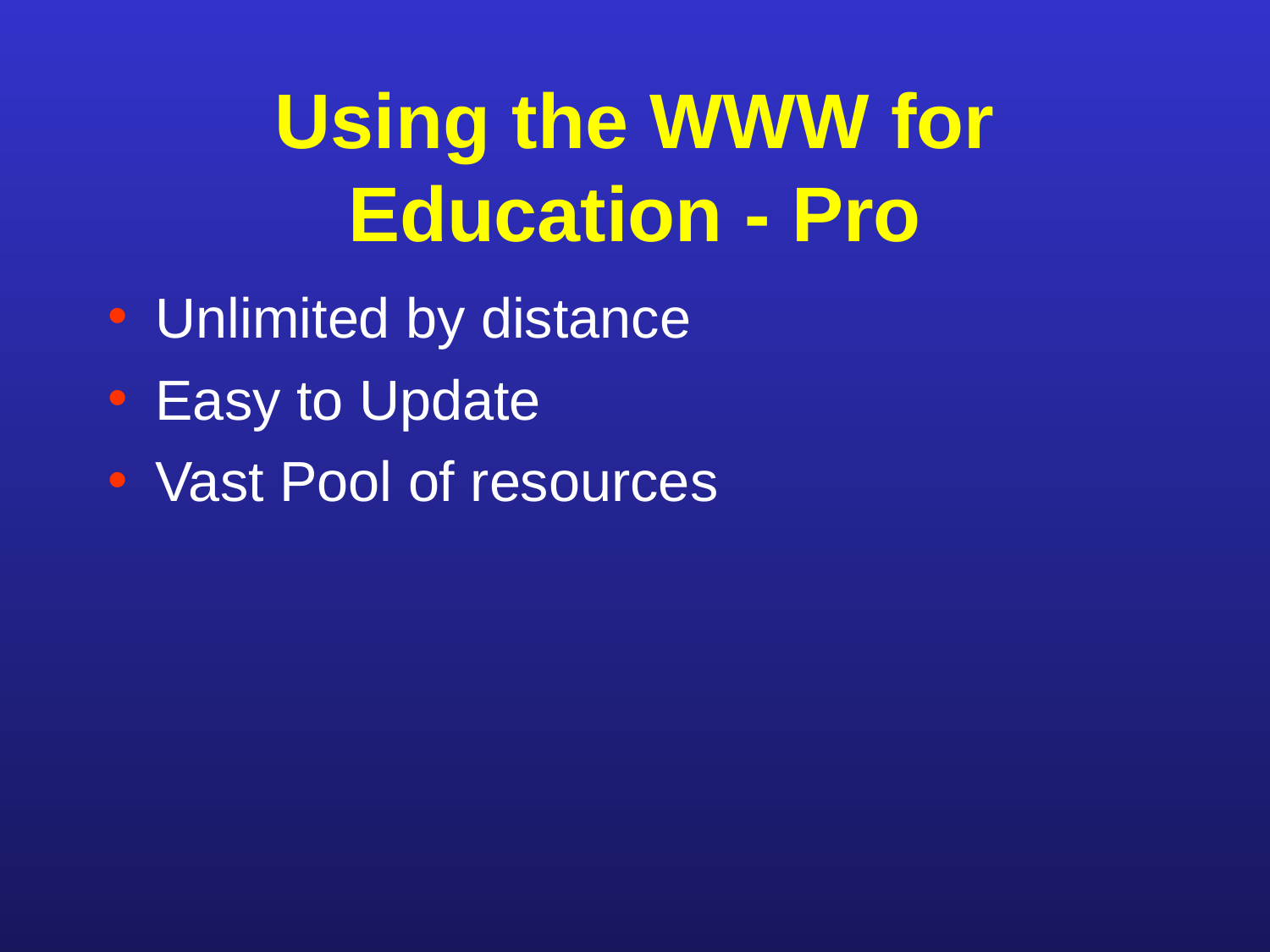

# Using the WWW for Education - Pro
Unlimited by distance
Easy to Update
Vast Pool of resources

## Slide 16
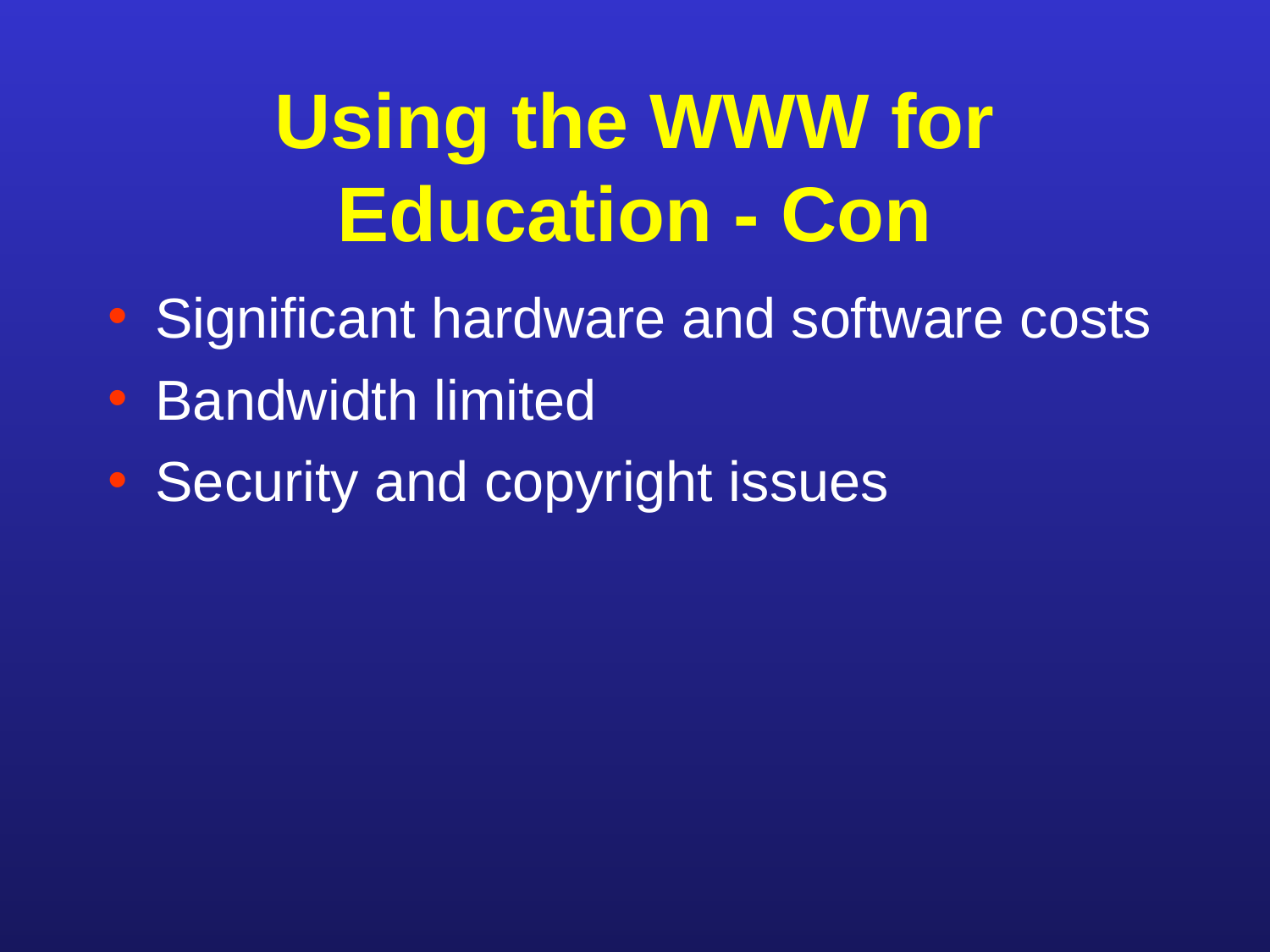

# Using the WWW for Education - Con
Significant hardware and software costs
Bandwidth limited
Security and copyright issues

## Slide 17
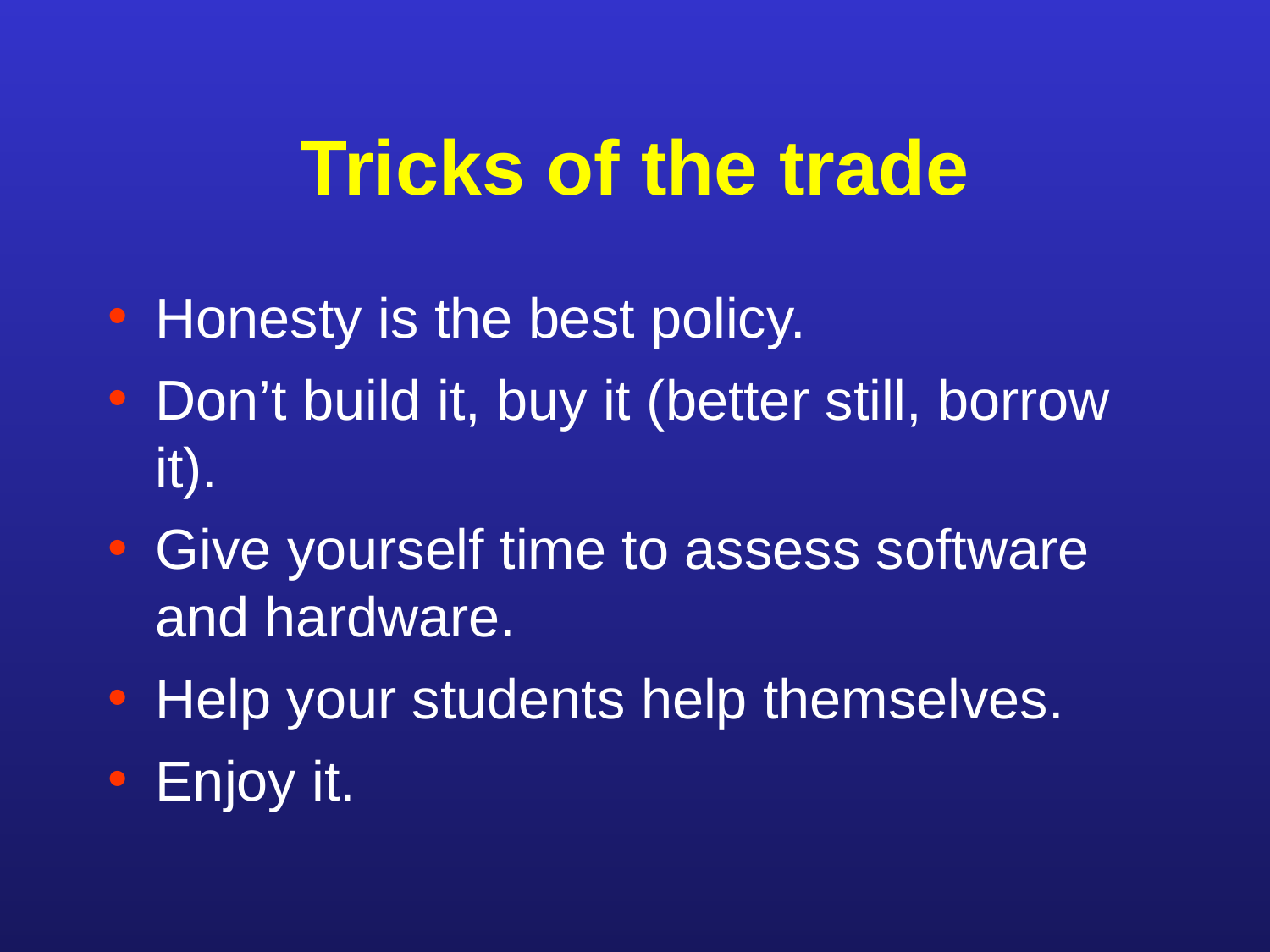

# Tricks of the trade
Honesty is the best policy.
Don’t build it, buy it (better still, borrow it).
Give yourself time to assess software and hardware.
Help your students help themselves.
Enjoy it.

## Slide 18
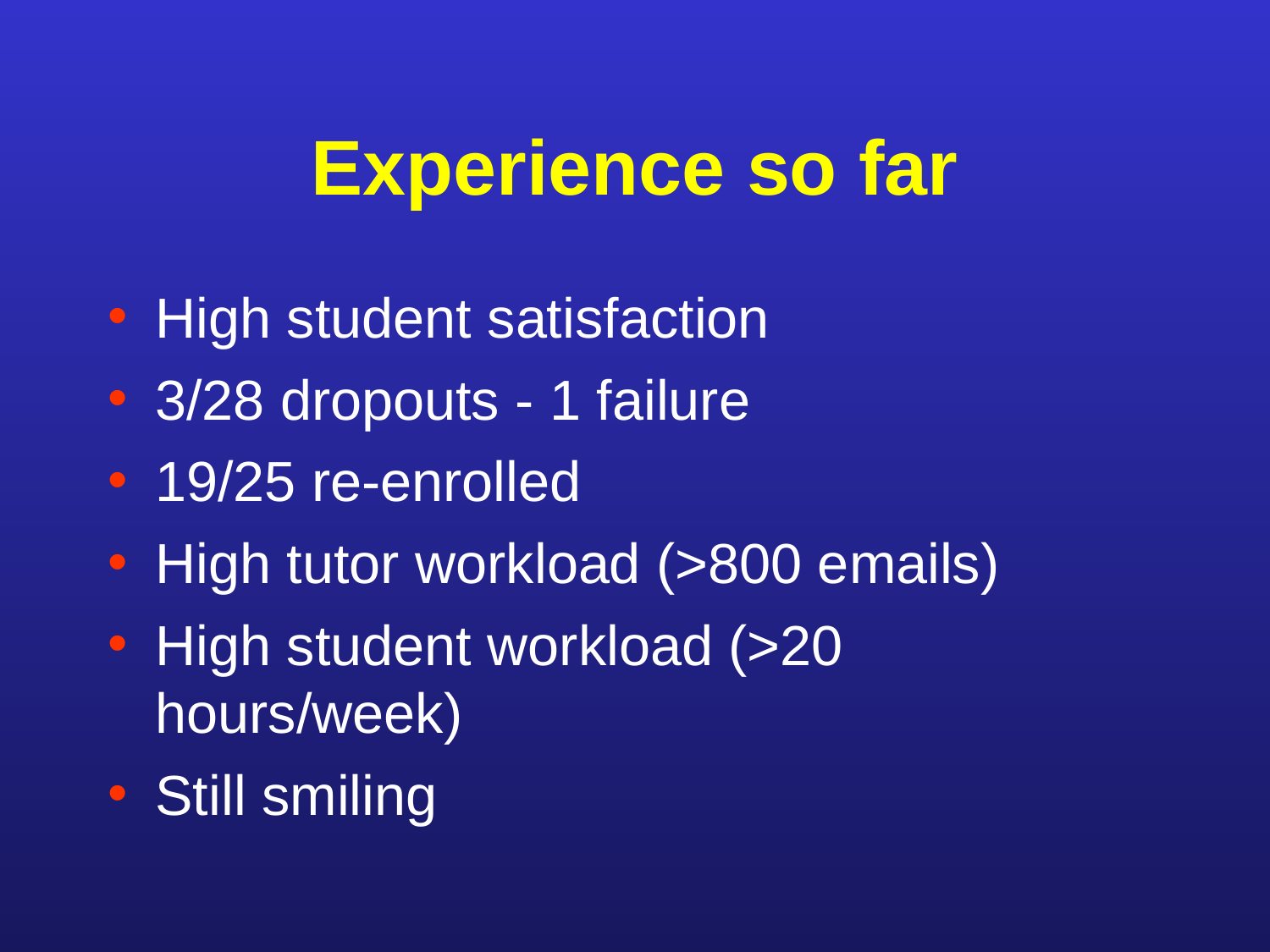

# Experience so far
High student satisfaction
3/28 dropouts - 1 failure
19/25 re-enrolled
High tutor workload (>800 emails)
High student workload (>20 hours/week)
Still smiling

## Slide 19
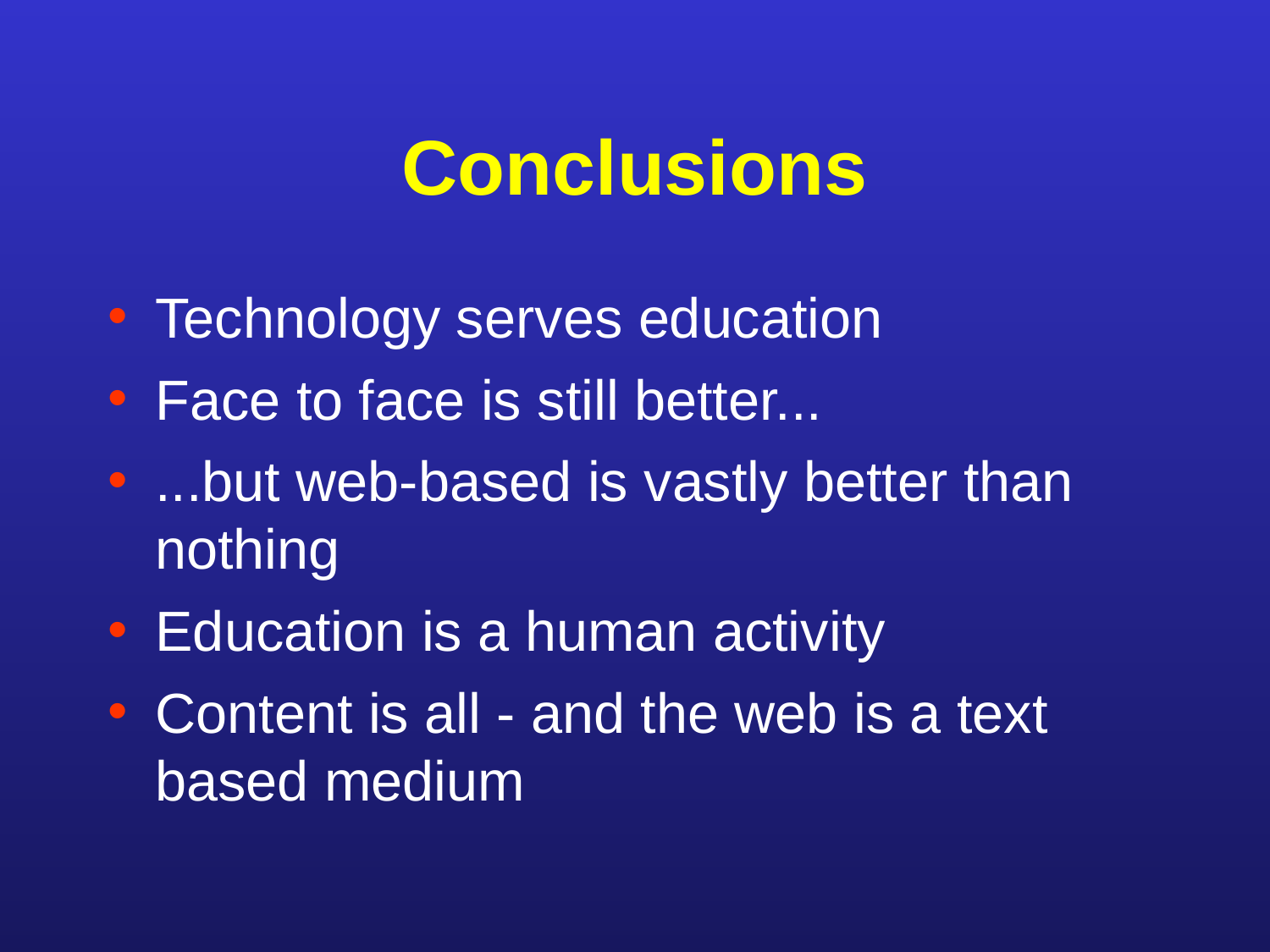

# Conclusions
Technology serves education
Face to face is still better...
...but web-based is vastly better than nothing
Education is a human activity
Content is all - and the web is a text based medium
